# Supplementary material for: Bioengineering developmentally inspired matrix vesicles as designer nanotherapeutics for bone regeneration
Source: Regen Biomater. 2026 Apr 20;13:rbag075. doi: 10.1093/rb/rbag075 (PMC13198379; doi:10.1093/rb/rbag075)
Supplement: rbag075_Supplementary_Data [file rbag075_supplementary_data.zip › rb-2025-777-33-40.pdf]

Supplementary figures

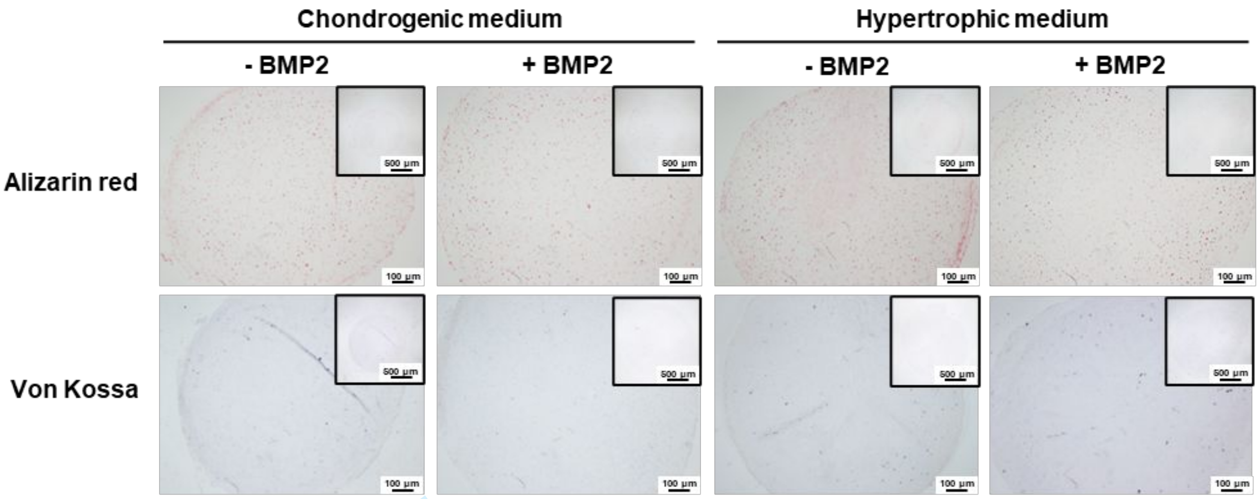

Supplementary Figure 1. Alizarin red and Von Kossa staining of hBMSC microtissues differentiated with or without BMP2 in chondrogenic or hypertrophic conditions.

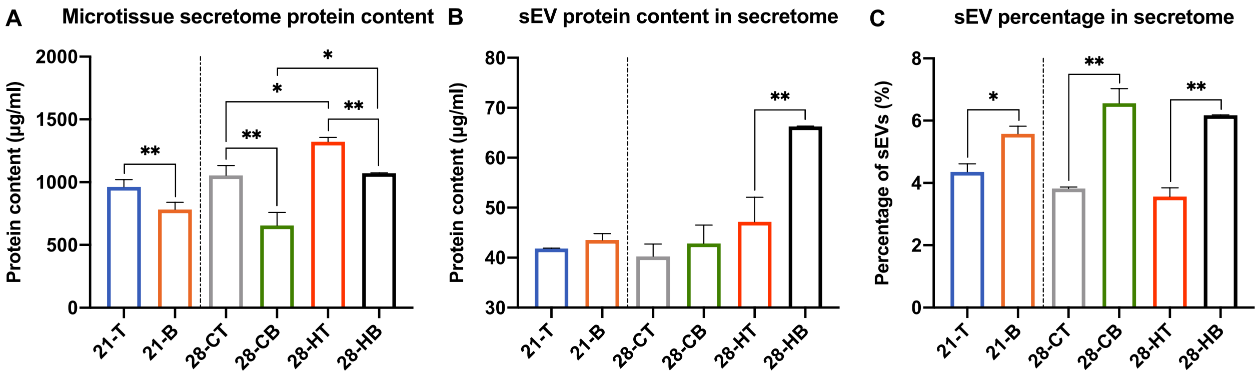

Supplementary Figure 2. Quantification of A) protein content, B) sEV content, and C) percentage of sEV in the microtissue-secretome at days 21 and 28. Data expressed as mean ± SD (N = 3). *P ≤ 0.05, **P ≤ 0.01 and ***P ≤ 0.001. CT = chondrogenic medium/-BMP2; CB = chondrogenic medium/+BMP2; HT = hypertrophic medium/-BMP2; HB = hypertrophic medium/+BMP2.

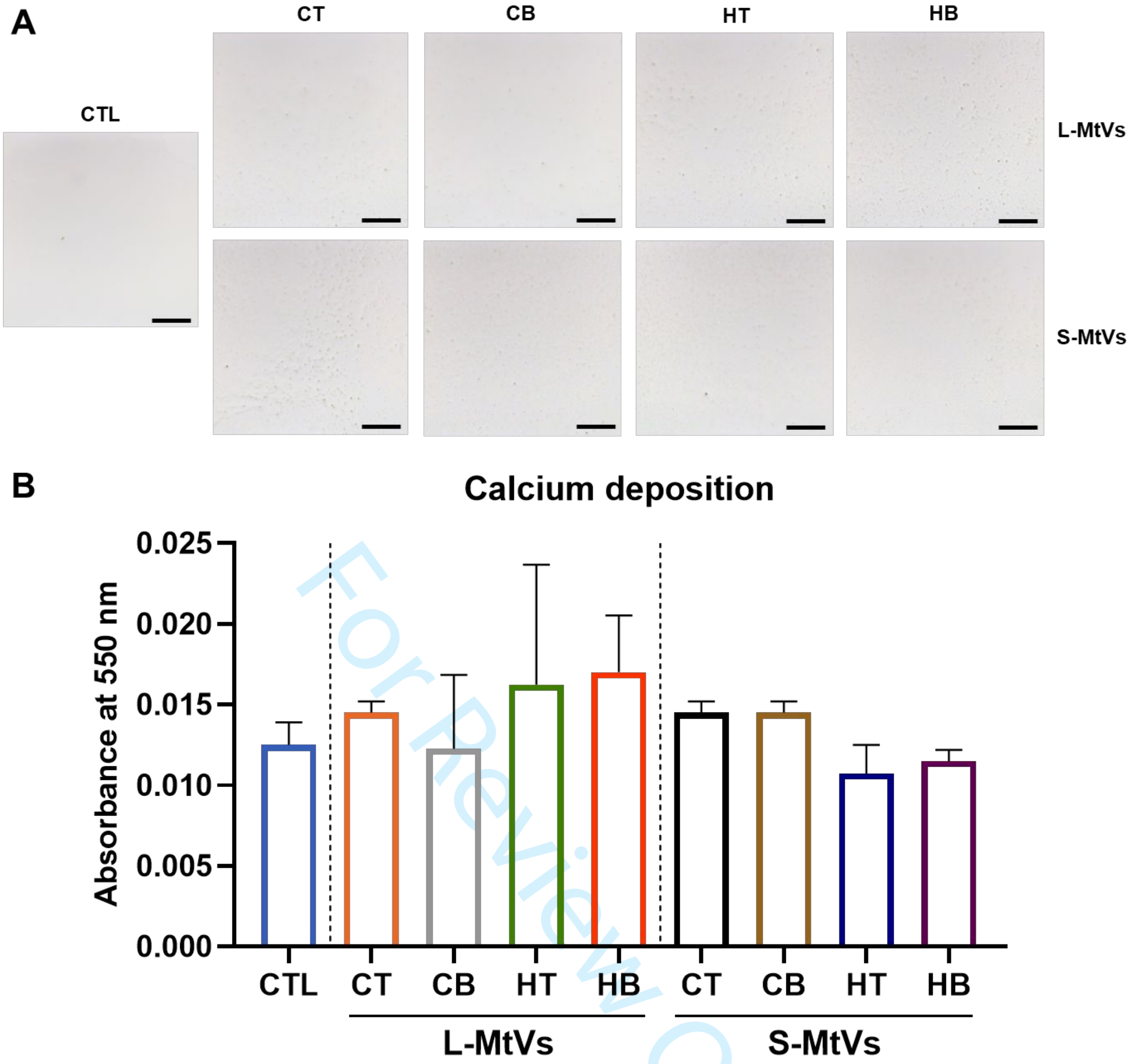

Supplementary Figure 3. The calcium content of MtVs-coated collagen surfaces. A) Alizarin red S staining for calcium deposition of MtV-functionalized collagen surfaces. Scale bar = 200 μm. B) Semi-quantification of calcium deposition. Data expressed as mean ± SD (N = 3). *P ≤ 0.05, **P ≤ 0.01 and ***P ≤ 0.001. CTL = control; CT = chondrogenic medium/-BMP2; CB = chondrogenic medium/+BMP2; HT = hypertrophic medium/-BMP2; HB = hypertrophic medium/+BMP2.

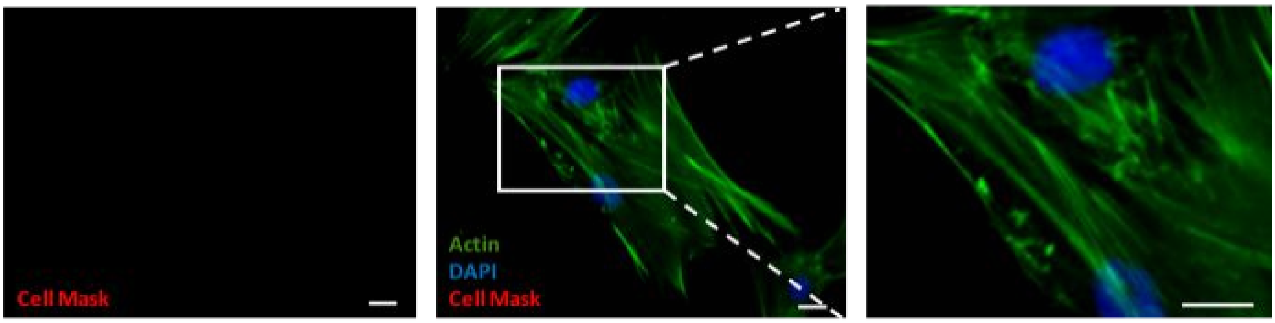

Supplementary Figure 4. Fluorescence images of non-MtV treated hBMSCs. Scale bar = 10 μm.

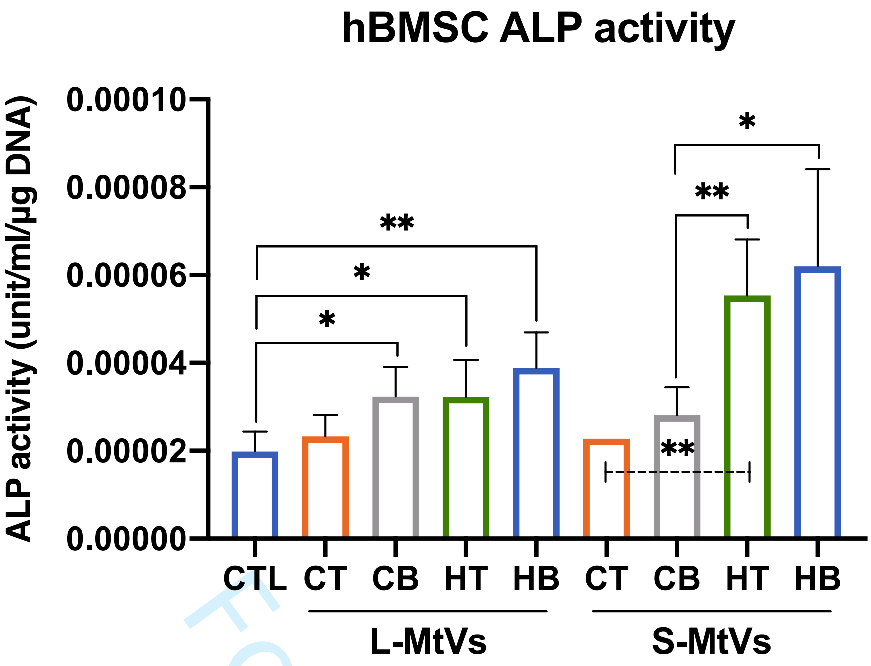

Supplementary Figure 5. ALP activity of MtV-treated hBMSCs in monolayer following 7 days of osteoinduction. Data expressed as mean ± SD (N= 3). *P ≤ 0.05, and **P ≤ 0.01. CT = chondrogenic medium/-BMP2; CB = chondrogenic medium/+BMP2; HT = hypertrophic medium/-BMP2; HB = hypertrophic medium/+BMP2.

For Review Only

References

1. Dimitriou R, Jones E, McGonagle D, Giannoudis PV. Bone regeneration: current concepts and future directions. *BMC Med* 2011;9:66.
2. Bahney CS, Zondervan RL, Allison P, Theologis A, Ashley JW, Ahn J, Miclau T, Marcucio RS, Hankenson KD. Cellular biology of fracture healing. *Journal of Orthopaedic Research* 2019;37:35-50.
3. Lewiecki EM, Ortendahl JD, Vanderpuye-Orgle J, Grauer A, Arellano J, Lemay J, Harmon AL, Broder MS, Singer AJ. Healthcare Policy Changes in Osteoporosis Can Improve Outcomes and Reduce Costs in the United States. *Jbmr Plus* 2019;3.
4. Calori GM, Mazza E, Colombo M, Ripamonti C. The use of bone-graft substitutes in large bone defects: any specific needs? *Injury* 2011;42 Suppl 2:S56-63.
5. Amini AR, Laurencin CT, Nukavarapu SP. Bone tissue engineering: recent advances and challenges. *Crit Rev Biomed Eng* 2012;40:363-408.
6. van Griensven M. Preclinical testing of drug delivery systems to bone. *Advanced Drug Delivery Reviews* 2015;94:151-164.
7. Mankin HJ, Hornicek FJ, Raskin KA. Infection in massive bone allografts. *Clinical Orthopaedics and Related Research* 2005:210-216.
8. Qu H, Fu H, Han Z, Sun Y. Biomaterials for bone tissue engineering scaffolds: a review. *RSC Adv* 2019;9:26252-26262.
9. James AW, LaChaud G, Shen J, Asatrian G, Nguyen V, Zhang X, Ting K, Soo C. A Review of the Clinical Side Effects of Bone Morphogenetic Protein-2. *Tissue Eng Part B Rev* 2016;22:284-97.
10. Bach DH, Park HJ, Lee SK. The Dual Role of Bone Morphogenetic Proteins in Cancer. *Molecular Therapy-Oncolytics* 2018;8:1-13.
11. Kim J, Tomida K, Matsumoto T, Adachi T. Spheroid culture for chondrocytes triggers the initial stage of endochondral ossification. *Biotechnol Bioeng* 2022;119:3311-3318.
12. Staubli F, Zhou Y, Vader P, Hofmann S, Bergsma JE, Gawlitta D, Man K. Harnessing extracellular vesicles for endochondral bone regeneration: Mechanisms and applications. *Acta Biomater* 2026.
13. Fu R, Liu CQ, Yan YX, Li QF, Huang RL. Bone defect reconstruction via endochondral ossification: A developmental engineering strategy. *Journal of Tissue Engineering* 2021;12.
14. Longoni A, Pennings I, Cuenca Lopera M, van Rijen MHP, Peperzak V, Rosenberg A, Levato R, Gawlitta D. Endochondral Bone Regeneration by Non-autologous Mesenchymal Stem Cells. *Front Bioeng Biotechnol* 2020;8:651.
15. Pelttari K, Winter A, Steck E, Goetzke K, Hennig T, Ochs BG, Aigner T, Richter W. Premature induction of hypertrophy during in vitro chondrogenesis of human mesenchymal stem cells correlates with calcification and vascular invasion after ectopic transplantation in SCID mice. *Arthritis and Rheumatism* 2006;54:3254-3266.

16. Drela K, Stanaszek L, Nowakowski A, Kuczynska Z, Lukomska B. Experimental Strategies of Mesenchymal Stem Cell Propagation: Adverse Events and Potential Risk of Functional Changes. *Stem Cells Int* 2019;2019:7012692.
17. Diederichs S, Shine KM, Tuan RS. The promise and challenges of stem cell-based therapies for skeletal diseases: stem cell applications in skeletal medicine: potential, cell sources and characteristics, and challenges of clinical translation. *Bioessays* 2013;35:220-30.
18. Jia YC, Zhu Y, Qiu S, Xu J, Chai YM. Exosomes secreted by endothelial progenitor cells accelerate bone regeneration during distraction osteogenesis by stimulating angiogenesis. *Stem Cell Research & Therapy* 2019;10.
19. Gnecchi M, He HM, Liang OD, Melo LG, Morello F, Mu H, Noiseux N, Zhang LN, Pratt RE, Ingwall JS, Dzau VJ. Paracrine action accounts for marked protection of ischemic heart by Akt-modified mesenchymal stem cells. *Nature Medicine* 2005;11:367-368.
20. Cunnane EM, Weinbaum JS, O'Brien FJ, Vorp DA. Future Perspectives on the Role of Stem Cells and Extracellular Vesicles in Vascular Tissue Regeneration. *Frontiers in Cardiovascular Medicine* 2018;5.
21. van Niel G, D'Angelo G, Raposo G. Shedding light on the cell biology of extracellular vesicles. *Nature Reviews Molecular Cell Biology* 2018;19:213-228.
22. Man K, Brunet MY, Jones MC, Cox SC. Engineered Extracellular Vesicles: Tailored-Made Nanomaterials for Medical Applications. *Nanomaterials* 2020;10.
23. Raposo G, Stoorvogel W. Extracellular vesicles: Exosomes, microvesicles, and friends. *Journal of Cell Biology* 2013;200:373-383.
24. Ferreira E, Porter RM. Harnessing extracellular vesicles to direct endochondral repair of large bone defects. *Bone Joint Res* 2018;7:263-273.
25. Wu D, Chen Q, Chen XJ, Han F, Chen Z, Wang Y. The blood-brain barrier: structure, regulation, and drug delivery. *Signal Transduction and Targeted Therapy* 2023;8.
26. Antimisiaris SG, Mourtas S, Marazioti A. Exosomes and Exosome-Inspired Vesicles for Targeted Drug Delivery. *Pharmaceutics* 2018;10.
27. Leung KS, Shirazi S, Cooper LF, Ravindran S. Biomaterials and Extracellular Vesicle Delivery: Current Status, Applications and Challenges. *Cells* 2022;11.
28. Pol F, Longoni A, Levato R, Gawlitta D, Man K. Extracellular vesicles in osteoimmunomodulation: Orchestrating immune-driven bone regeneration. *Int J Biol Macromol* 2026;338:149614.
29. Gil Izquierdo S, Fernandez Pilar A, Rios JL, Lim KS, Toh WS, Liu C, Gimona M, Gawlitta D, Man K. Advances in extracellular vesicle-based nanomedicine for regenerative orthopaedics. *J Nanobiotechnology* 2025;24:36.
30. Boyan BD, Asmussen NC, Lin Z, Schwartz Z. The Role of Matrix-Bound Extracellular Vesicles in the Regulation of Endochondral Bone Formation. *Cells* 2022;11.
31. Anderson HC. Electron microscopic studies of induced cartilage development and calcification. *J Cell Biol* 1967;35:81-101.
32. Bonucci E. Fine structure of early cartilage calcification. *J Ultrastruct Res* 1967;20:33-50.
33. Bottini M, Mebarek S, Anderson KL, Strzelecka-Kiliszek A, Bozycki L, Simao AMS, Bolean M, Ciancaglini P, Pikula JB, Pikula S, Magne D, Volkmann N, Hanein D, Millán JL, Buchet R. Matrix vesicles from chondrocytes and osteoblasts: Their biogenesis, properties, functions and biomimetic models. *Biochimica Et Biophysica Acta-General Subjects* 2018;1862:532-546.
34. Lin Z, McClure MJ, Zhao JJ, Ramey AN, Asmussen N, Hyzy SL, Schwartz Z, Boyan BD. MicroRNA Contents in Matrix Vesicles Produced by Growth Plate Chondrocytes are Cell Maturation Dependent. *Scientific Reports* 2018;8.
35. Kirsch T, Nah HD, Shapiro IM, Pacifici M. Regulated production of mineralization-competent matrix vesicles in hypertrophic chondrocytes. *Journal of Cell Biology* 1997;137:1149-1160.
36. Nahar NN, Missana LR, Garimella R, Tague SE, Anderson HC. Matrix vesicles are carriers of bone morphogenetic proteins (BMPs), vascular endothelial growth factor (VEGF), and noncollagenous matrix proteins. *Journal of Bone and Mineral Metabolism* 2008;26:514-519.
37. Bessueille L, Briolay A, Como J, Mebarek S, Mansouri C, Gleizes M, El Jamal A, Buchet R, Dumontet C, Matera EL, Mornet E, Millan JL, Fonta C, Magne D. Tissue-nonspecific alkaline phosphatase is an anti-inflammatory nucleotidase. *Bone* 2020;133:115262.
38. Su GY, Zhang DM, Li TT, Pei T, Yang J, Tu SS, Liu SJ, Ren J, Zhang YJ, Duan MM, Yang XR, Shen Y, Zhou CC, Xie J, Liu XH. Annexin A5 derived from matrix vesicles protects against osteoporotic bone loss via mineralization. *Bone Research* 2023;11.
39. Laurencin CT, El-Amin SF. Xenotransplantation in orthopaedic surgery. *Journal of the American Academy of Orthopaedic Surgeons* 2008;16:4-8.
40. Petrus-Reurer S, Romano M, Howlett S, Jones JL, Lombardi G, Saeb-Parsy K. Immunological considerations and challenges for regenerative cellular therapies. *Communications Biology* 2021;4.
41. Gawlitta D, van Rijen MHP, Schrijver EJM, Alblas J, Dhert WJA. Hypoxia Impedes Hypertrophic Chondrogenesis of Human Multipotent Stromal Cells. *Tissue Engineering Part A* 2012;18:1957-1966.
42. Man K, Mekhileri N, Lim K, Jiang LH, Woodfield T, Yang XB. MI192 induced epigenetic reprogramming enhances the therapeutic efficacy of human bone marrows stromal cells for bone regeneration. *Bone* 2021:116138.
43. de Silva L, Longoni A, Staubli F, Nurmohamed S, Duits A, Rosenberg A, Gawlitta D. Bone Regeneration in a Large Animal Model Featuring a Modular Off-the-Shelf Soft Callus Mimetic. *Adv Healthc Mater* 2023;12:e2301717.

For Review Only

44. Chen NX, O'Neill KD, Chen XM, Moe SM. Annexin-Mediated Matrix Vesicle Calcification in Vascular Smooth Muscle Cells. *Journal of Bone and Mineral Research* 2008;23:1798-1805.
45. Man K, Brunet MY, Louth S, Robinson TE, Fernandez-Rhodes M, Williams S, Federici AS, Davies OG, Hoey DA, Cox SC. Development of a Bone-Mimetic 3D Printed Ti6Al4V Scaffold to Enhance Osteoblast-Derived Extracellular Vesicles' Therapeutic Efficacy for Bone Regeneration. *Front Bioeng Biotechnol* 2021;9:757220.
46. Man K, Brunet MY, Fernandez-Rhodes M, Williams S, Heaney LM, Gethings LA, Federici A, Davies OG, Hoey D, Cox SC. Epigenetic reprogramming enhances the therapeutic efficacy of osteoblast-derived extracellular vesicles to promote human bone marrow stem cell osteogenic differentiation. *J Extracell Vesicles* 2021;10:e12118.
47. Moghassemi S, Dadashzadeh A, Sousa MJ, Vlieghe H, Yang J, Leon-Felix CM, Amorim CA. Extracellular vesicles in nanomedicine and regenerative medicine: A review over the last decade. *Bioact Mater* 2024;36:126-156.
48. De Jong OG, Van Balkom BW, Schiffelers RM, Bouten CV, Verhaar MC. Extracellular vesicles: potential roles in regenerative medicine. *Front Immunol* 2014;5:608.
49. Ansari S, de Wildt BWM, Vis MAM, de Korte CE, Ito K, Hofmann S, Yuana Y. Matrix Vesicles: Role in Bone Mineralization and Potential Use as Therapeutics. *Pharmaceuticals* 2021;14.
50. Wu MR, Wu SL, Chen W, Li YP. The roles and regulatory mechanisms of TGF-β and BMP signaling in bone and cartilage development, homeostasis and disease. *Cell Research* 2024;34:101-123.
51. Kobayashi T, Lyons KM, McMahon AP, Kronenberg HM. BMP signaling stimulates cellular differentiation at multiple steps during cartilage development. *Proceedings of the National Academy of Sciences of the United States of America* 2005;102:18023-18027.
52. Seo HS, Serra R. Deletion of Tgfbr2 in Prx1-cre expressing mesenchyme results in defects in development of the long bones and joints. *Developmental Biology* 2007;310:304-316.
53. Spagnoli A, O'Rear L, Chandler RL, Granero-Molto F, Mortlock DP, Gorska AE, Weis JA, Longobardi L, Chytil A, Shimer K, Moses HL. TGF-β signaling is essential for joint morphogenesis. *Journal of Cell Biology* 2007;177:1105-1117.
54. Zhang DH, Schwarz EM, Rosier RN, Zuscik MJ, Puzas JE, O'Keefe RJ. ALK2 functions as a BMP type I receptor and induces Indian hedgehog in chondrocytes during skeletal development. *Journal of Bone and Mineral Research* 2003;18:1593-1604.
55. Yoon BS, Pogue R, Ovchinnikov DA, Yoshii I, Mishina Y, Behringer RR, Lyons KM. BMPs regulate multiple aspects of growth-plate chondrogenesis through opposing actions on FGF pathways. *Development* 2006;133:4667-4678.
56. Yan JY, Li J, Hu J, Zhang L, Wei CG, Sultana N, Cai XQ, Zhang WJ, Cai CL. Smad4 deficiency impairs chondrocyte hypertrophy via the Runx2 transcription factor in mouse skeletal development. *Journal of Biological Chemistry* 2018;293:9162-9175.
57. Furumatsu T, Tsuda M, Taniguchi N, Tajima Y, Asahara H. Smad3 induces chondrogenesis through the activation of SOX9 via CREB-binding protein/p300 recruitment. *Journal of Biological Chemistry* 2005;280:8343-8350.
58. Longoni A, Utomo L, Robinson A, Levato R, Rosenberg AJWP, Gawlitta D. Acceleration of Bone Regeneration Induced by a Soft-Callus Mimetic Material. *Advanced Science* 2022;9.
59. Cancedda FD, Gentili C, Manduca P, Cancedda R. Hypertrophic Chondrocytes Undergo Further Differentiation in Culture. *Journal of Cell Biology* 1992;117:427-435.
60. Roach HI, Erenpreisa J, Aigner T. Osteogenic Differentiation of Hypertrophic Chondrocytes Involves Asymmetric Cell Divisions and Apoptosis. *Journal of Cell Biology* 1995;131:483-494.
61. Scotti C, Tonnarelli B, Papadimitropoulos A, Scherberich A, Schaeren S, Schauerte A, Lopez-Rios J, Zeller R, Barbero A, Martin I. Recapitulation of endochondral bone formation using human adult mesenchymal stem cells as a paradigm for developmental engineering. *Proceedings of the National Academy of Sciences of the United States of America* 2010;107:7251-7256.
62. Man KY, Eisenstein NM, Hoey DA, Cox SC. Bioengineering extracellular vesicles: smart nanomaterials for bone regeneration. *Journal of Nanobiotechnology* 2023;21.
63. Marsell R, Einhorn TA. The biology of fracture healing. *Injury* 2011;42:551-5.
64. Welsh JA, Goberdhan DCI, O'Driscoll L, Buzas EI, Blenkiron C, Bussolati B, Cai HJ, Di Vizio D, Driedonks TAP, Erdbrügger U, Falcon-Perez JM, Fu QL, Hill AF, Lenassi M, Lim SK, Mahoney MG, Mohanty S, Möller A, Nieuwland R, ..., Consortium M. Minimal information for studies of extracellular vesicles (MISEV2023): From basic to advanced approaches. *Journal of Extracellular Vesicles* 2024;13.
65. Man K, Barroso IA, Brunet MY, Peacock B, Federici AS, Hoey DA, Cox SC. Controlled Release of Epigenetically-Enhanced Extracellular Vesicles from a GelMA/Nanoclay Composite Hydrogel to Promote Bone Repair. *Int J Mol Sci* 2022;23.
66. Qin YH, Wang L, Gao ZL, Chen GY, Zhang CQ. Bone marrow stromal/stem cell-derived extracellular vesicles regulate osteoblast activity and differentiation in vitro and promote bone regeneration in vivo. *Scientific Reports* 2016;6.
67. Crescitelli R, Lasser C, Lotvall J. Isolation and characterization of extracellular vesicle subpopulations from tissues. *Nat Protoc* 2021;16:1548-1580.
68. Balcerzak M, Radisson J, Azzar G, Farlay D, Boivin G, Pikula S, Buchet R. A comparative analysis of strategies for isolation of matrix vesicles. *Anal Biochem* 2007;361:176-82.
69. Anderson HC, Cecil R, Sajdera SW. Calcification of rachitic rat cartilage in vitro by extracellular matrix vesicles. *Am J Pathol* 1975;79:237-54.

For Review Only

70. Kirsch T, Wuthier RE. Stimulation of Calcification of Growth-Plate Cartilage Matrix Vesicles by Binding to Type-Ii and Type-X Collagens. *Journal of Biological Chemistry* 1994;269:11462-11469.
71. Schmidt JR, Kliemt S, Preissler C, Moeller S, von Bergen M, Hempel U, Kalkhof S. Osteoblast-released Matrix Vesicles, Regulation of Activity and Composition by Sulfated and Non-sulfated Glycosaminoglycans. *Molecular & Cellular Proteomics* 2016;15:558-572.
72. Mackie EJ, Ahmed YA, Tatarczuch L, Chen KS, Mirams M. Endochondral ossification: How cartilage is converted into bone in the developing skeleton. *International Journal of Biochemistry & Cell Biology* 2008;40:46-62.
73. Anderson HC. Matrix vesicles and calcification. *Curr Rheumatol Rep* 2003;5:222-6.
74. Miao DS, Scutt A. Histochemical localization of alkaline phosphatase activity in decalcified bone and cartilage. *Journal of Histochemistry & Cytochemistry* 2002;50:333-340.
75. Jang WG, Kim EJ, Kim DK, Ryoo HM, Lee KB, Kim SH, Choi HS, Koh JT. BMP2 Protein Regulates Osteocalcin Expression via Runx2-mediated Gene Transcription. *Journal of Biological Chemistry* 2012;287:905-915.
76. Boyan BD, Schwartz Z, Swain LD, Carnes DL, Zislis T. Differential Expression of Phenotype by Resting Zone and Growth Region Costochondral Chondrocytes Invitro. *Bone* 1988;9:185-194.
77. Kirsch T, Harrison G, Golub EE, Nah HD. The roles of annexins and types II and X collagen in matrix vesicle-mediated mineralization of growth plate cartilage. *Journal of Biological Chemistry* 2000;275:35577-35583.
78. Wu LNY, Genge BR, Lloyd GC, Wuthier RE. Collagen-Binding Proteins in Collagenase-Released Matrix Vesicles from Cartilage - Interaction between Matrix Vesicle Proteins and Different Types of Collagen. *Journal of Biological Chemistry* 1991;266:1195-1203.
79. Man KB, M. Y.; Federici, A. S.; Hoey, D. A.; Cox, S. C.;. An ECM-Mimetic Hydrogel to Promote the Therapeutic Efficacy of Osteoblast-Derived Extracellular Vesicles for Bone Regeneration *Frontiers in Bioengineering and Biotechnology* 2022;10.
80. Sung BH, Parent CA, Weaver AM. Extracellular vesicles: Critical players during cell migration. *Developmental Cell* 2021;56:1861-1874.
81. Lee YJ, Park M, Kim HY, Kim JK, Kim WK, Lim SC, Kang KW. Circulating small extracellular vesicles promote proliferation and migration of vascular smooth muscle cells via AXL and MerTK activation. *Acta Pharmacologica Sinica* 2023;44:984-998.
82. Joshi BS, de Beer MA, Giepmans BNG, Zuhorn IS. Endocytosis of Extracellular Vesicles and Release of Their Cargo from Endosomes. *ACS Nano* 2020;14:4444-4455.
83. Gurung S, Perocheau D, Touramanidou L, Baruteau J. The exosome journey: from biogenesis to uptake and intracellular signalling. *Cell Communication and Signaling* 2021;19.
84. Liu SH, Liu Y, Jiang LB, Li Z, Lee S, Liu CS, Wang J, Zhang J. Recombinant human BMP-2 accelerates the migration of bone marrow mesenchymal stem cells via the CDC42/PAK1/LIMK1 pathway in vitro and in vivo. *Biomaterials Science* 2019;7:362-372.
85. Kronenberg HM. Developmental regulation of the growth plate. *Nature* 2003;423:332-336.
86. Alini M, Marriott A, Chen T, Abe S, Poole AR. A novel angiogenic molecule produced at the time of chondrocyte hypertrophy during endochondral bone formation. *Developmental Biology* 1996;176:124-132.
87. Finkenzeller G, Hager S, Stark GB. Effects of bone morphogenetic protein 2 on human umbilical vein endothelial cells. *Microvascular Research* 2012;84:81-85.
88. Wiley DM, Jin SW. Bone Morphogenetic Protein functions as a context-dependent angiogenic cue in vertebrates. *Seminars in Cell & Developmental Biology* 2011;22:1012-1018.
89. Cao LY, Wang J, Hou J, Xing WL, Liu CS. Vascularization and bone regeneration in a critical sized defect using 2-,6-sulfated chitosan nanoparticles incorporating BMP-2. *Biomaterials* 2014;35:684-698.
90. Zhu HL, Z.; Luan, Q.; Yang, Y.; Chen, M.; Liu, X.; Wang, J.; Man, K.; Zhang. J.;. Angiogenesis-promoting composite TPMS bone tissue engineering scaffold for mandibular defect regeneration. *International Journal of Bioprinting* 2023;10.
91. Kuttappan S, Mathew D, Jo JI, Tanaka R, Menon D, Ishimoto T, Nakano T, Nair SV, Nair MB, Tabata Y. Dual release of growth factor from nanocomposite fibrous scaffold promotes vascularisation and bone regeneration in rat critical sized calvarial defect. *Acta Biomaterialia* 2018;78:36-47.
92. Saidak Z, Le Henaff C, Azzi S, Marty C, Da Nascimento S, Sonnet P, Marie PJ. Wnt/beta-catenin signaling mediates osteoblast differentiation triggered by peptide-induced alpha5beta1 integrin priming in mesenchymal skeletal cells. *J Biol Chem* 2015;290:6903-12.
93. Dong J, Xu X, Zhang Q, Yuan Z, Tan B. The PI3K/AKT pathway promotes fracture healing through its crosstalk with Wnt/beta-catenin. *Exp Cell Res* 2020;394:112137.
94. Cheng BF, Feng X, Gao YX, Jian SQ, Liu SR, Wang M, Xie YF, Wang L, Feng ZW, Yang HJ. Neural Cell Adhesion Molecule Regulates Osteoblastic Differentiation Through Wnt/beta-Catenin and PI3K-Akt Signaling Pathways in MC3T3-E1 Cells. *Front Endocrinol (Lausanne)* 2021;12:657953.
95. Dean DD, Schwartz ZV, Muniz OE, Gomez R, Swain LD, Howell DS, Boyan BD. Matrix vesicles contain metalloproteinases that degrade proteoglycans. *Bone Miner* 1992;17:172-6.
96. D'Angelo M, Billings PC, Pacifici M, Leboy PS, Kirsch T. Authentic matrix vesicles contain active metalloproteases (MMP). a role for matrix vesicle-associated MMP-13 in activation of transforming growth factor-beta. *J Biol Chem* 2001;276:11347-53.

For Review Only

97. Shibuya M. Vascular Endothelial Growth Factor (VEGF) and Its Receptor (VEGFR) Signaling in Angiogenesis: A Crucial Target for Anti- and Pro-Angiogenic Therapies. *Genes Cancer* 2011;2:1097-105.
98. ten Dijke P, Goumans MJ, Pardali E. Endoglin in angiogenesis and vascular diseases. *Angiogenesis* 2008;11:79-89.
99. Rivera LB, Bradshaw AD, Brekken RA. The regulatory function of SPARC in vascular biology. *Cell Mol Life Sci* 2011;68:3165-73.
100. Liu T, Guevara OE, Warburton RR, Hill NS, Gaestel M, Kayyali US. Regulation of vimentin intermediate filaments in endothelial cells by hypoxia. *Am J Physiol Cell Physiol* 2010;299:C363-73.
101. Kiraly N, Thalwieser Z, Fonodi M, Csortos C, Boratko A. Dephosphorylation of annexin A2 by protein phosphatase 1 regulates endothelial cell barrier. *IUBMB Life* 2021;73:1257-1268.
102. Ling Q, Jacovina AT, Deora A, Febbraio M, Simantov R, Silverstein RL, Hempstead B, Mark WH, Hajjar KA. Annexin II regulates fibrin homeostasis and neoangiogenesis in vivo. *J Clin Invest* 2004;113:38-48.
103. Bouter A, Gounou C, Berat R, Tan S, Gallois B, Granier T, d'Estaintot BL, Poschl E, Brachvogel B, Brisson AR. Annexin-A5 assembled into two-dimensional arrays promotes cell membrane repair. *Nat Commun* 2011;2:270.
104. Graifer D, Malygin A, Shefer A, Tamkovich S. Ribosomal Proteins as Exosomal Cargo: Random Passengers or Crucial Players in Carcinogenesis? *Adv Biol (Weinh)* 2025;9:e2400360.
105. Hong H, Lin C, Fang M, Liu J, Hsu HC, Chang CJ, Wang H. Proteomic analysis of exosomal proteins associated with bone healing speed in a rat tibial fracture model. *Biomed Chromatogr* 2024;38:e5846.
106. Ochkasova A, Arbuzov G, Malygin A, Graifer D. Two "Edges" in Our Knowledge on the Functions of Ribosomal Proteins: The Revealed Contributions of Their Regions to Translation Mechanisms and the Issues of Their Extracellular Transport by Exosomes. *Int J Mol Sci* 2023;24.
107. Eyre DR. Collagens and cartilage matrix homeostasis. *Clin Orthop Relat Res* 2004:S118-22.
108. Moursi AM, Damsky CH, Lull J, Zimmerman D, Doty SB, Aota S, Globus RK. Fibronectin regulates calvarial osteoblast differentiation. *J Cell Sci* 1996;109 (Pt 6):1369-80.
109. Shainer R, Kram V, Kilts TM, Li L, Doyle AD, Shainer I, Martin D, Simon CG, Jr., Zeng-Brouwers J, Schaefer L, Young MF, Genomics, Computational Biology C. Biglycan regulates bone development and regeneration. *Front Physiol* 2023;14:1119368.
110. Yang F, Luo P, Ding H, Zhang C, Zhu Z. Collagen type V a2 (COL5A2) is decreased in steroid-induced necrosis of the femoral head. *Am J Transl Res* 2018;10:2469-2479.
111. Saadeh PB, Mehrara BJ, Steinbrech DS, Dudziak ME, Greenwald JA, Luchs JS, Spector JA, Ueno H, Gittes GK, Longaker MT. Transforming growth factor-beta1 modulates the expression of vascular endothelial growth factor by osteoblasts. *Am J Physiol* 1999;277:C628-37.
112. Kato Y, Lewalle JM, Baba Y, Tsukuda M, Sakai N, Baba M, Kobayashi K, Koshika S, Nagashima Y, Frankenne F, Noel A, Foidart JM, Hata RI. Induction of SPARC by VEGF in human vascular endothelial cells. *Biochem Biophys Res Commun* 2001;287:422-6.
113. Jin M, Zhang J, Sun Y, Liu G, Wei X. ANXA5: related mechanisms of osteogenesis and additional biological functions. *Front Cell Dev Biol* 2025;13:1553683.
114. Umbrecht-Jenck E, Demais V, Calco V, Bailly Y, Bader MF, Chasserot-Golaz S. S100A10-mediated translocation of annexin-A2 to SNARE proteins in adrenergic chromaffin cells undergoing exocytosis. *Traffic* 2010;11:958-71.
115. Xu Y, Tan H, Liu K, Wen C, Pang C, Liu H, Xu R, Li Q, He C, Nandakumar KS, Zhou C. Targeted inhibition of ATP5B gene prevents bone erosion in collagen-induced arthritis by inhibiting osteoclastogenesis. *Pharmacol Res* 2021;165:105458.
116. Dai J, Escara-Wilke J, Keller JM, Jung Y, Taichman RS, Pienta KJ, Keller ET. Primary prostate cancer educates bone stroma through exosomal pyruvate kinase M2 to promote bone metastasis. *J Exp Med* 2019;216:2883-2899.
117. Xie Y, Guan Q, Guo J, Chen Y, Yin Y, Han X. Hydrogels for Exosome Delivery in Biomedical Applications. *Gels* 2022;8.
118. Xie H, Wang Z, Zhang L, Lei Q, Zhao A, Wang H, Li Q, Cao Y, Jie Zhang W, Chen Z. Extracellular Vesicle-functionalized Decalcified Bone Matrix Scaffolds with Enhanced Pro-angiogenic and Pro-bone Regeneration Activities. *Sci Rep* 2017;7:45622.
119. Zhai M, Zhu Y, Yang M, Mao C. Human Mesenchymal Stem Cell Derived Exosomes Enhance Cell-Free Bone Regeneration by Altering Their miRNAs Profiles. *Adv Sci (Weinh)* 2020;7:2001334.
120. Cheng Y, Dong X, Shi J, Wu G, Tao P, Ren N, Zhao Y, Li F, Wang Z. Immunomodulation with M2 macrophage-derived extracellular vesicles for enhanced titanium implant osseointegration under diabetic conditions. *Mater Today Bio* 2025;30:101385.

For Review Only

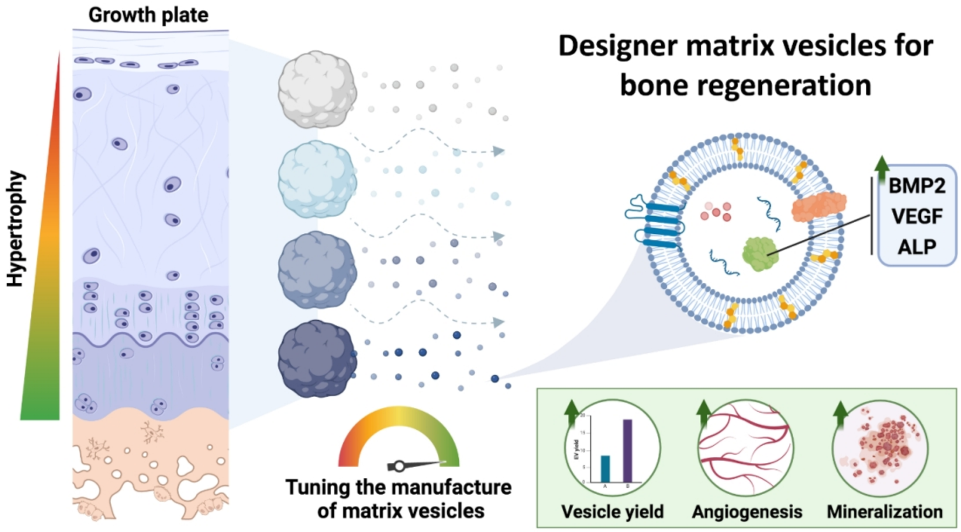

169x96mm (220 x 220 DPI)
